# Aspects of the Application of Cavity Enhanced Spectroscopy to Nitrogen Oxides Detection

**DOI:** 10.3390/s130607570

**Published:** 2013-06-10

**Authors:** Jacek Wojtas, Janusz Mikolajczyk, Zbigniew Bielecki

**Affiliations:** Institute of Optoelectronics, Military University of Technology, 2 Gen. S. Kaliskiego St., Warsaw 00-908, Poland; E-Mails: jmikolajczyk@wat.edu.pl (J.M.); zbielecki@wat.edu.pl (Z.B.)

**Keywords:** CEAS, CRDS, absorption spectroscopy, laser spectroscopy, nitrogen oxides detection, NO_x_ detection, explosives detection

## Abstract

This article presents design issues of high-sensitive laser absorption spectroscopy systems for nitrogen oxides (NO_x_) detection. Examples of our systems and their investigation results are also described. The constructed systems use one of the most sensitive methods, cavity enhanced absorption spectroscopy (CEAS). They operate at different wavelength ranges using a blue—violet laser diode (410 nm) as well as quantum cascade lasers (5.27 μm and 4.53 μm). Each of them is configured as a one or two channel measurement device using, e.g., time division multiplexing and averaging. During the testing procedure, the main performance features such as detection limits and measurements uncertainties have been determined. The obtained results are 1 ppb NO_2_, 75 ppb NO and 45 ppb N_2_O. For all systems, the uncertainty of concentration measurements does not exceed a value of 13%. Some experiments with explosives are also discussed. A setup equipped with a concentrator of explosives vapours was used. The detection method is based either on the reaction of the sensors to the nitrogen oxides directly emitted by the explosives or on the reaction to the nitrogen oxides produced during thermal decomposition of explosive vapours. For TNT, PETN, RDX and HMX a detection limit better than 1 ng has been achieved.

## 1. Introduction

Nitrogen oxides are compounds of nitrogen and oxygen. They play a significant role in many different fields of technology and science. They are important greenhouse gases and one of the compounds responsible for the generation of acid rains. Moreover, many explosive materials are sources of these gases as the result of their decomposition products. This phenomenon can be applied in explosives detection using spectroscopy [1]. Additionally, nitrous oxide is used as an anaesthetic, especially in dentistry and minor surgery, and nitric oxide as a biomarker of respiratory inflammation or asthma in human breath diagnosis [2].

Nowadays, many techniques of laser spectroscopy are applied to NO_x_ detection. They make it possible to detect the presence of a substance based on the phenomena of scattering, emission or absorption of optical radiation [3–5]. In Table 1 examples of the techniques applying lasers to NO_x_ detection are listed. Their detection limit varies from ppt-level (e.g., integrated cavity output spectroscopy—ICOS [6]) to hundreds of ppm (e.g., differential optical absorption spectroscopy—DOAS [7]).

**Figure 1.**
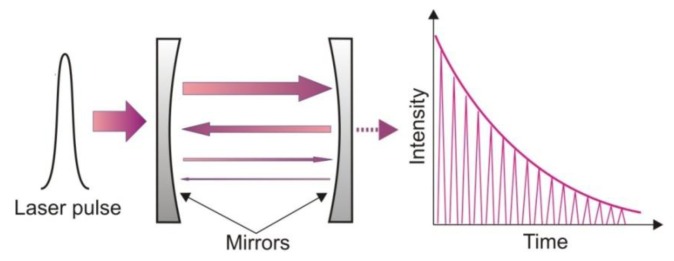
Cavity ring down spectroscopy idea.

Involving conventional absorption spectroscopy, cavity ring down spectroscopy (CRDS) provides a better detection limit. The idea of the CRDS is shown in Figure 1. The main element of the setup is an optical cavity with a high quality factor. In the cavity, two concave mirrors with a very high reflectivity *R* are mounted. This construction provides a long optical path, even up to several kilometres [20].

During the detection procedure, a light pulse is injected into the cavity through one of the mirrors. Inside the cavity multiple reflections are observed. After each reflection, leakage radiation from the cavity is registered with the photodetector. If the laser spectrum and the absorption spectrum of the gas filling the cavity are appropriately matched, the cavity quality will decrease. This phenomenon is registered by the photodetector. Based on the photodetector response, the absorption coefficient and concentration of the gas can be determined.

**Figure 2.**
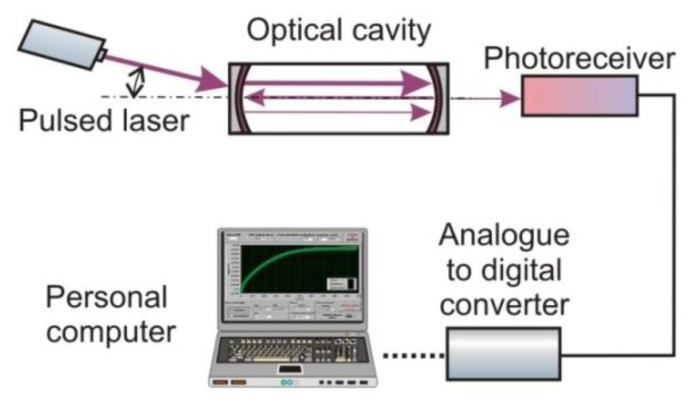
The scheme of the CEAS setup.

A modification of the CRDS is a cavity enhanced absorption spectroscopy (CEAS) proposed in 1998 by Engeln *et al.* [21]. The operation idea of the two methods is similar, but there is a difference related to the alignment of a laser beam and the cavity as well as to the modes structure. In CEAS the radiation is injected at a very small angle in respect to the cavity axis (Figure 2). It results in the formation of a dense structure of weak modes, which can overlap each other [22]. Sometimes, in addition to the output mirror, a piezoelectric-driven mount is used to modulate the cavity length. Thanks to this, the establishment of a constant mode structure within the cavity is minimized [23].

The weak mode structure makes the entire system much less sensitive to instability in both the cavity and laser spectrum. Additionally, due to off-axis alignment, the interference by the feedback radiation from the cavity mirror is eliminated. For example, in case of nitrogen dioxide detection CEAS sensors attain a detection limit at the of sub-ppb level (*i.e.*, about 10^−9^ cm^−1^) [24,25]. Therefore, this method creates the opportunity to develop a portable optoelectronic sensor to detection of trace concentrations of nitrogen oxides.

**Figure 3.**
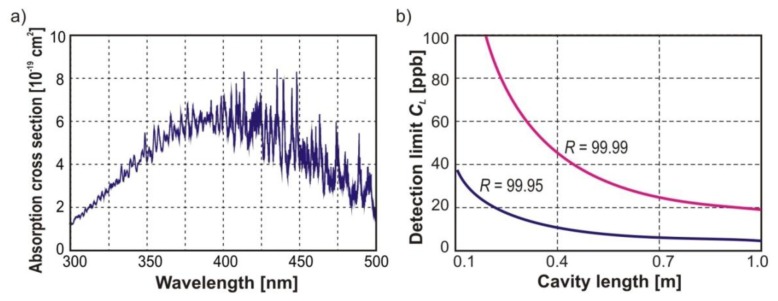
NO_2_ absorption spectrum (**a**) and dependence of the detection limit on the cavity length and the reflectivity of mirrors *R* (**b**).

In the visible spectral range, observation of NO_x_ molecules can be done from electronic transitions. These transitions are characterized by broad absorption spectra providing a relatively large mean absorption cross section. Therefore, in this wavelength range, broadband multimode lasers can be applied. In the case of nitrogen dioxide, the absorption spectrum has a band in the 395–430 nm range with a mean cross section of about 6 × 10^−19^ cm^2^ (Figure 3(a)). Examples of developed nitrogen dioxide sensor assemblies are briefly described in Section 4 and discussed in detail in several papers [26–28]. The sensitivities of the designed NO_2_ sensors reach the level of 0.1 ppb and strictly depend on, e.g., cavity length, mirror reflectivity (*R*) (Figure 3(b)).

**Figure 4.**
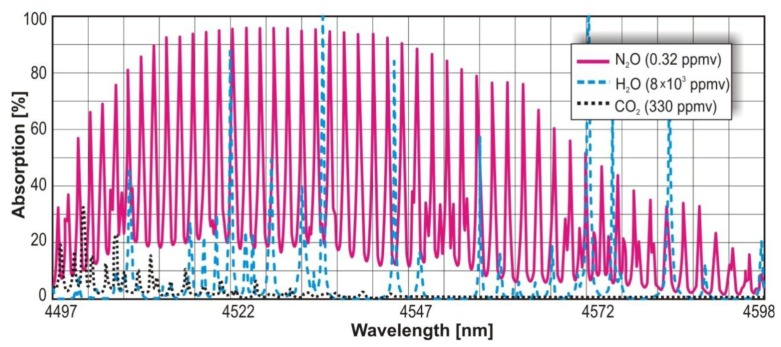
N_2_O absorption spectrum.

**Figure 5.**
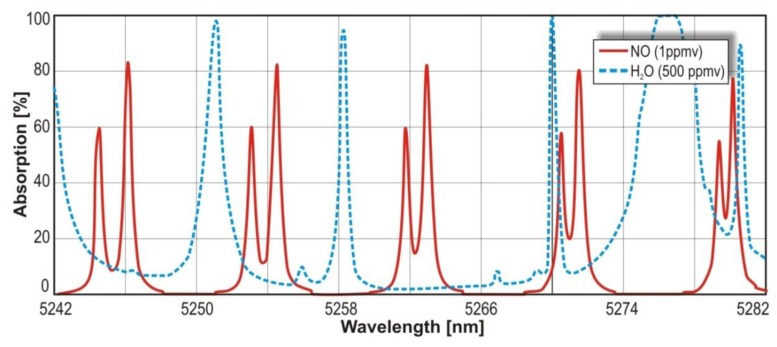
NO absorption spectrum.

In the infrared range, the N_2_O absorption cross section reaches the value 3.9 × 10^−18^ cm^2^ at the wavelength range 4.51 μm –4.56 μm. For NO, the cross section of 0.7 × 10^−18^ cm^2^ at spectral range of 5.24 μm –5.28 μm is observed. In Figures 4 and 5, maxima of the nitric oxide and nitrous oxide absorption spectrum are shown. The molecules absorption coefficient is the product of their absorption cross section and their concentration. The absorption spectra of the gases were found by use of HITRAN database [29].

The value of absorption cross section and parameters of the sensors make it possible to reach a ppb level detection limit [30]. Additionally, in the described wavelength ranges there is no significant interference from absorption lines of other atmospheric gases (e.g., CO_2_, H_2_O). For H_2_O, the interference can be also minimized with the use of low compound pressure and special particle filters or driers.

## 2. Visible and Mid-Infrared Lasers

**Figure 6.**
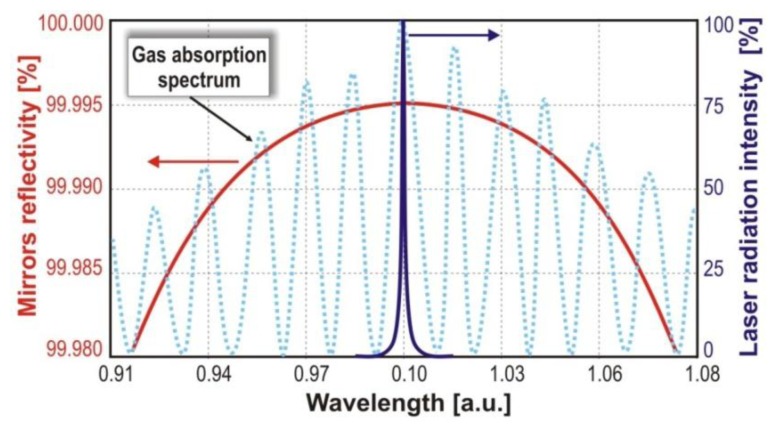
Illustration of matching the laser emission wavelength and cavity mirrors reflectivity.

In laser absorption spectroscopy, the appropriate matching of the laser emission wavelength to the absorption spectrum of investigated gas (Figure 6) should be taken into account. The lasers should be characterized by, e.g., high power and single-mode of emitted radiation, compact construction, long lifetimes, stable parameters in cw and pulse mode of operation. Additional, cavity enhanced methods require the appropriate selection of cavity mirrors. Their reflectivity should have a maximum value at wavelength corresponding to the absorption line of the investigated gas.

In the UV and VIS spectral range, gas lasers and solid-state lasers could be applied [31]. They are characterized with good beam parameters and high power. Their main disadvantages are complex power supply systems, necessity of efficient cooling (usually liquid), significant dimensions and high price. In laser absorption spectroscopy dye lasers are applied as well. They give the possibility to work in a wide range of wavelengths, depending on the type of dye. However, semiconductor lasers are applied more often. They make it possible to design compact and cheap sensor systems. Currently, rapid progress in the field of semiconductor lasers [32–37] has been observed. For instance, commercially available blue or near UV nitride-based laser diodes are characterized by high power, narrow spectrum (below 1 nm), long lifetime and with low power consumption. This type of lasers is very suitable for spectroscopy applications. For example Zybin *et al.* [38] specified 34 chemical elements displaying strong absorption lines reachable with GaN lasers diode (blue light). Precise atomic spectroscopy employing these lasers was already demonstrated. For example, a few constructions of a highly sensitive detector of the NO_2_ compound have been reported [39–42].

Infrared spectroscopy is also an important tool for the detection and identification of trace concentration gases (molecules). Currently available systems are suitable for detection at the level of ppm, ppb and also of ppt. Such performance was achieved by using rovibrational spectroscopy in the wavelength range from 3.5 μm to 24 μm. Currently, there are several sources of radiation which are able to operate in the mid-infrared range, e.g., laser diodes with lead-salt and antimonide compounds, sources with parametric frequency conversion (PFC), tunable solid state-lasers and quantum cascade (QC) lasers.

In practice, lead-salt diode lasers require a low temperature of operation and they emit radiation with a maximum power of 500 μW. In the case of PFC, the mid-IR generation is obtained using nonlinear materials. The main technologies are difference frequency generation (DFG) and optical parametric oscillation (OPO) [43]. These systems are characterized by complex construction, high price and demand very stable operating conditions. There are also portable DFGs. However, these instruments have a limited spectral bandwidth, extremely low efficiency of conversion and low levels of output power (from hundreds of microwatts to a few milliwatts) [44].

An important step in the development of IR spectroscopy was the discovery in 1994 of quantum cascade lasers. Nowadays, QC lasers are very suitable radiation sources for experiments with cavity enhanced methods because of the integrated design, narrow emission lines and high luminosity. Moreover, their emission wavelength can be easy tuned to the maxima of N_2_O and NO absorption cross section. Present techniques, which are used for broad wavelength tuning, include external cavity (EC) tuning, array setup of distributed feedback lasers, voltage tuning, *etc*. For the first two techniques, the gain spectral width of the laser chips is the main determinant of the maximum tuning ranges. Some different techniques to achieve a broad gain spectrum have been demonstrated, such as heterogeneous quantum cascade lasers. However, an increase in broad gain width usually leads to a decrease in a level of peak gain. Results of this effect are: lower value of both radiation power and energy efficiency of the lasers [45].

Currently, there are multiple types of QC lasers constructions that are able to be applied to spectroscopy. It could be, e.g., Fabry-Perot lasers (FP-QC), distributed feedback lasers (DFB-QC), EC-QC lasers or lasers with built-in integrating difference frequency generation structure (DFG-QC). FP lasers are characterized by higher powers of the radiation (of the order even 120 W) and multimode radiation structure. The porous structure is fabricated towards the sides of the QCL waveguide [46]. That is why high order transverse modes are suppressed and the fundamental mode reaches the threshold first. This laser structure makes it possible to obtain a temperature tuning rate of 0.49 nm/K.

In DFB-QC lasers, the precise control of the emission wavelength and a single mode operation is possible. It is achieved using a distributed feedback resonator. These lasers are characterized by lower power and narrower spectral lines of radiation. The tuning range of the spectrum is narrow and reaches values of 4 cm^−1^ for current driving and about 20 cm^−1^ for temperature setting [44]. In room temperature with continuous wave (cw) operation, wall plug efficiency (WPE) of 27% has been achieved. For spectral or spatial beam shaping, some additional technologies such as: one dimensional distributed feedback, photonic crystal distributed feedback or ring cavity surface emitting QC lasers are designed [47,48]. For example, a quantum cascade laser with a photonic crystal emits at the wavelength of 7.6 μm with a temperature of 80 °C. In this structure, single-mode operation with a high signal-to-noise ratio and narrow beam divergence of 6.2° could be reached. A high peak power of 630 mW at 20 °C and more than 160 mW at 60 °C was also registered. The obtained performance makes this laser a very promising tool in long-wave infrared spectroscopy [49].

A special group of QC lasers (EC-QCL) characterized by a broad spectrum of radiation that was also designed (even more than 500 cm^−1^) was mentioned [50,51]. The lasers are dedicated to operate with external optical systems shaping the emission spectrum, e.g., cavities, gratings [52,53]. For instance, lasing wavelength of such lasers is able to be tuned in the range of 180 cm^−1^ at 8.6 μm and FWHM lasing line up to 7 × 10^−4^ cm^−1^ [44,45], and with output power of up to 300 mW [11,54,55].

Radiation sources which employ multi-section laser geometry have also been developed. Fabrication of QC lasers emitting radiation at several wavelengths simultaneously makes it possible to achieve a larger tuning range [56]. For example, a two-wavelength laser emitting at 5.2 μm and 8.0 μm and an ultra-broadband laser covering all wavelengths from 6.0 μm to 8.0 μm have been developed by Gmachl *et al.* [57]. In addition, QC laser arrays have been also designed. These structures give the possibility of tuning the spectrum up to 200 cm^−1^ with a linewidth (FWHM) of ~0.01 cm^−1^ and ~0.0001 cm ^−1^ for pulse or cw operation mode respectively [43]. At present, commercial DFB-QC lasers are available, the lasing wavelength of which could be selected from the range of 3 μm−5 μ m. Some technology limitations in the case of fabrication of QC lasers operated at shorter wavelengths are observed [58]. Practically, there are two techniques to obtain a laser operating at a shorter wavelength range. The first one requires working at much lower temperatures. The cooling is due to high current density [59]. The second way is to design interband cascade lasers (ICL) [60]. This type of lasers can radiate in a wavelength range of 3 μm−4 μm at room temperature, reaching an average power of above 10 mW.

In the case of long-wave-range, there are no such restrictions and for infrared laser spectroscopy the wavelength of 24 μm determines the upper range of the spectrum. Above this value, THz technology conventionally begins.

Additionally, it should be noted that there is ongoing work on the development of QCL technologies. The one of them is to retune the spectrum using external optical radiation [61]. It is reported that reversible tuning by about 6 cm^−1^ was achieved. In Table 2 some parameters of selected types of quantum cascade lasers are listed.

## 3. Signal Processing Unit

**Figure 7.**
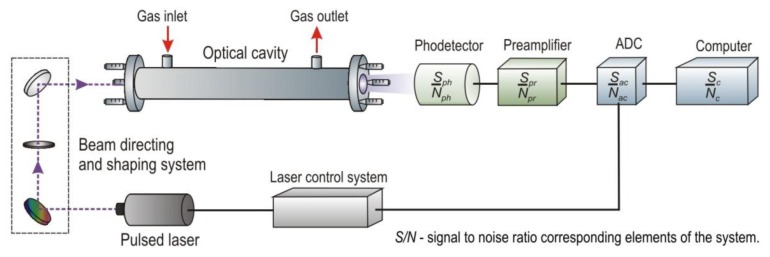
Block diagram of NO_x_ sensor.

The optical signal from the cavity is registered with a photoreceiver, the operating spectrum of which should be matched to the selected absorption line of the gas. It is usually characterized by high gain, wide dynamic range, appropriately selected signal waveband and low noises. [68]. Next, the signal from the photoreceiver is digitized using an analogue-to-digital converter (ADC). Special computer software provides processing of the measuring data and gas concentration determination. A scheme of a signal processing in the cavity enhanced sensor is presented in Figure 7.

### 3.1. Photodetector

In photoreceiver construction, there are two types of photodetectors: thermal (e.g., thermopile, bolometer and pyroelectric—Py) and photon (e.g., photomultiplier, photodiode and photoresistor). The first group is characterized by an almost constant curve of spectral responsivity, but high response times and low detectivity make these detectors useless in cavity enhanced spectroscopy. Therefore, photon photodetectors with both higher responsivity and higher speed find application in such detection systems. To achieve better performance they can be extra cooled, especially in the IR range of the wavelength.

In the case of ultraviolet (UV), visible (VIS) and near infrared (NIR) region the most popular are photomultiplier tubes (PMT’s). They are characterized by high gain, high speed and low dark current. Because of PMT high resistance, transimpedance preamplifiers are usually used to amplify the signal from PMT. They are also characterized by a wide dynamic range [69].

**Figure 8.**
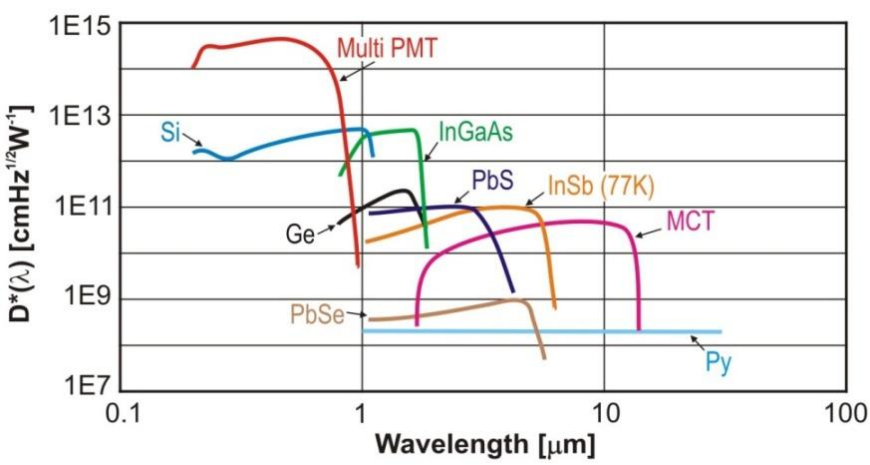
Example of photodetectors detectivity.

For the detection of MIR radiation, the most popular are InSb photodiodes and PbSe photoresistors as well as MCT photoresistors and photodiodes (Figure 8). These detectors are characterized by high speed, low noise and excellent uniformity, linearity and stability. They can operate in the photovoltaic mode. To achieve the best properties, InSb detectors have to operate in low temperatures, such as 77 K. PbSe photoconductive detectors are able to detect radiation in the wavelength range from 2.0 μm to 6.0 μm. The main virtues are low cost, high efficiency and compact design. Thanks to thermoelectric cooling (TEC) it is possible to provide higher sensitivity, wider spectral range and higher stability of temperature using [70−72]. In Figure 8 detectivity characteristics of multi-anode photomultiplier tubes (Multi PMT) are presented as well. There are also available types of PMT’s with spectral response up to 1.7 μm, e.g., H10330A or R5509 [69].

In the case of MCT detectors, spectral detection range is defined by the hetero-structure materials and their sizes (e.g., mole fraction of cadmium to mercury). In the case of a photoconductive detector, its detectivity is changed with the active area, field of view, temperature and a bias current. The increase in the active area causes a reduction of the signal bandwidth and an increase in the noise. In practice, the relative growth in the surface size is achieved by using an immersion lens. In comparison to PbSe detectors, MCTs are characterized by higher dynamics and detectivity, lower bias voltage and shorter response time. They can be also cooled by thermoelectric or liquid nitrogen systems. MCT detectors can also be designed for operation in photovoltaic mode. Application of cooling increases the dark resistance, reducing the noise level and improving detectivity. Nowadays, a wide range of commercial MCT photodiodes operating at room temperature using one-, two-, three- or four TEC stages are offered. The cooling makes it possible to control sensitivity and cut-off wavelength. An alternative detectors for the middle and long wavelength IR region are also quantum well infrared photodetector (QWIPs) and super-lattice (SL) photodiodes. QWIP cannot compete with MCT photodiode because have relatively low quantum efficiencies, typically less than 10%. The spectral response band is also narrow for this detector. In comparison, the SL structures provide high responsivity, as already reached with MCT. But for these detectors, fabrication of high quality photodiodes is very important technological challenge. The detectors are typically DC coupled with transimpedance preamplifier or AC coupled with voltage ones. The AC coupling is characterized with lower value of *1/f* noise than DC ones. The main features of the selected detectors are listed in Table 3.

In summary, both photoconductive MCT detectors and photovoltaic MCT detectors are very attractive for infrared laser spectroscopy. Compact detection modules with MCT detectors that use monolithic optical immersion technology are available, integrated with optimized amplifiers and TEC cooling. They offer high detectivity (about 10^12^ cmHz^1/2^W^−1^) and wide frequency bandwidth (up to 1 GHz) [71,72].

### 3.2. Photoreceiver with Photomultiplier Tube

**Figure 9.**
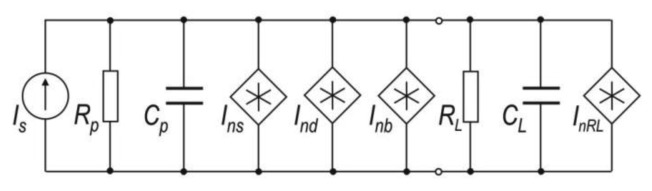
PMT equivalent scheme.

To determine the signal-to-noise ratio of the photoreceiver, the PMT equivalent scheme should be analyzed (Figure 9). In this scheme, *I_s_* represents the useful signal, *R_p_* and *C_p_* are the resistance and capacitance of the photomultiplier respectively, *I_ns_* is the current shot noise of useful signal, *I_nd_* is current shot noise of anode dark current, *I_nb_* is current noise from background radiation and *I_nRL_* is the thermal current noise of load with parameters of *R_L_* and *C_L_* [73].

Taking into consideration the described noise sources, the formula for PMT signal-to-noise ratio determination is given by [74]:

(1) $$\frac{S_{\text{ph}}}{N_{\text{ph}}}=\frac{I_{s}^{2}}{I_{\text{ns}}^{2}+I_{\text{nd}}^{2}+I_{\text{nb}}^{2}+I_{\text{nRL}}^{2}}$$

Assuming that during experiments background noise can be eliminated, a photoemission process is described by the *Poisson* model and all stages of PMT have the same gain, then the signal-to-noise ratio equals:

(2) $$\frac{S_{\text{ph}}}{N_{\text{ph}}}=\frac{{\left(P_{s}\cdot S_{p}\cdot G_{p}\right)}^{2}}{2\;q\;\Delta \;f_{n}\;(G_{p}\;S_{p}\;P_{s}+I_{\text{nd}}\;)\frac{\delta }{\delta -1}+\frac{4\;k\;T_{o}\;\Delta \;f_{n}}{R_{L}}},$$

where *P_s_* is the power of optical radiation, *G_p_* is the PMT gain, *S_p_* is the photocathode sensitivity, *q* is the electron charge, *Δf_n_* is the noise bandwidth, *δ* is one stage of the PMT gain, *k* is the Boltzmann constant and *T_0_* is the temperature [75].

The noise bandwidth can be determined from the formula:

(3) $$\Delta f_{n}=\frac{\pi }{2}\Delta f_{3dB}\approx \frac{1}{4R_{L}(C_{L}+C_{P})},$$

where *Δf_3dB_* represents 3 dB frequency bandwidth.

**Figure 10.**
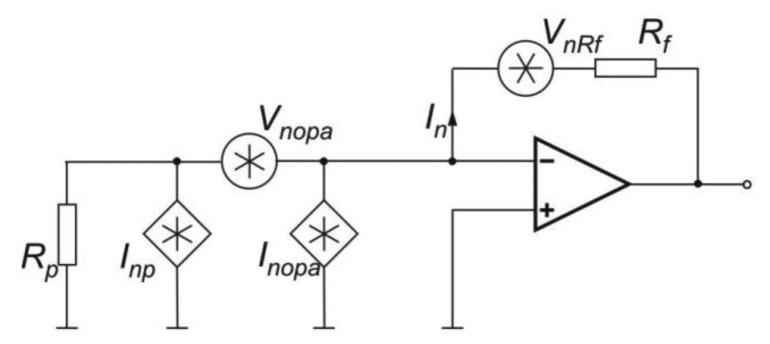
Equivalent scheme of the first stage of the photoreceiver.

For the PMT, the best preamplifier configuration is a transimpedance preamplifier. The *SNR* determination of the photoreceiver requires analyses of its equivalent scheme (Figure 10).

In the scheme, signals sources of *V_nopa_*, *I_nopa_* and *I_np_* represents both voltage and current noise of the operation amplifier and current noise of PMT respectively.

The total current noise *I_nt_* is given by:

(4) $$I_{\text{nt}}^{2}=I_{\text{np}}^{2}+{\left(V_{\text{nopa}}\frac{R_{p}+R_{f}}{R_{p}R_{f}}\right)}^{2}+I_{\text{nopa}}^{2}+{\left(\frac{V_{\text{nRf}}}{R_{f}}\right)}^{2}$$

where *V_nRf_* equals:

(5) $$V_{\text{nRf}}^{2}=4kT_{o}R_{f}\Delta f_{n}$$

and photoreceiver signal-noise-ratio is expressed by the equation:

(6) $$\frac{S_{\text{pr}}}{N_{\text{pr}}}=\frac{I_{s}^{2}}{I_{\text{nt}}^{2}}$$

The photoreceiver output signal is fed to an A/D converter and the noise level increases of the factor of:

(7) $$V_{\text{nadc}}^{2}=\frac{{\delta_{\text{adc}}}^{2}}{12}$$

where *δ_adc_* is the quantization step.

Summarized, the *SNR* of the signal processing unit consists of PMT, preamplifier and ADC converter is given by:

(8) $$\frac{S_{\text{ac}}}{N_{\text{ac}}}=\frac{{\left(P_{s}S_{p}G_{p}\;\right)}^{2}\cdot R_{f}^{2}}{\gamma R_{f}^{2}\Delta f_{n}+V_{\text{nadc}}^{2}}$$

where:

(9) $$\gamma =2q(G_{p}S_{p}P_{s}+I_{da})\frac{\delta }{\delta -1}+{\left(V_{\text{nopa}}\frac{R_{p}+R_{f}}{R_{p}\cdot R_{f}}\right)}^{2}+I_{\text{nopa}}^{2}+\frac{4kT_{o}}{R_{f}}$$

### 3.3. Photoreceiver with a MCT Photodiode

**Figure 11.**
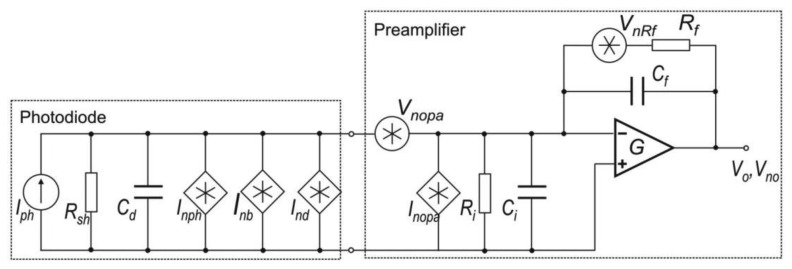
Scheme of the photoreceiver with a photodiode.

Figure 11 shows the noise equivalent scheme of a MCT photodiode with a transimpedance preamplifier.

In the scheme *I_ph_* represents the photocurrent and *I_nph_*, *I_nd_* and *I_nb_* are the shot noises caused by photocurrent, dark current and background current respectively [76].

In this circuit, the value of the photodiode load resistance depends on the feedback resistance *R_f_* and the amplifier open-loop gain *G*. Settings value of resistance *R_f_*, the influence on the level of both the preamplifier output signal and its noise is observed.

Assuming the equivalent photoreceiver noise equals the square root of sum of each component noise squares, the signal-to-noise ratio can be expressed by [77]:

(10) $$\frac{S_{\text{pr}}}{N_{\text{pr}}}=\frac{I_{\text{ph}}^{2}}{\left(I_{\text{nph}}^{2}+I_{\text{nd}}^{2}+I_{\text{nb}}^{2}+I_{\text{nopa}}^{2}+\frac{4kT\Delta f}{R_{f}}\right)+{\left(V_{\text{nopa}}\frac{R_{\text{eq}}^{2}}{1+\omega^{2}\tau_{\text{eq}}^{2}}\right)}^{2}}$$

where:

(11) $$R_{\text{eq}}=\frac{R_{f}R_{\text{sh}}}{R_{f}+R_{\text{sh}}},\;\tau_{\text{eq}}=R_{\text{eq}}(C_{f}\;+C_{d}\;)$$

Furthermore, if background current and all shot noise currents are negligibly, the *SNR* of signal processing unit consisting in a photodiode, preamplifier and AD converter can be determined from the equation:

(12) $$\frac{S_{\text{ac}}}{N_{\text{ac}}}=\frac{{\left(R_{i}P_{s}\right)}^{2}}{{\left(\frac{R_{i}{\left(A\Delta f\right)}^{1/2}}{D^{*}}\right)}^{2}+{I_{\text{nopa}}}^{2}+\frac{4kT\Delta f}{R_{f}}+{\left(\frac{V_{\text{nopa}}}{R_{f}}\right)}^{2}+V_{\text{nadc}}^{2}}$$

where *R_i_*—photodiode current responsivity, *A*—detector active area.

The *SNR* of the CEAS setup can be additionally improved by the use some advanced detection methods, *i.e.*, coherent averaging [78]. This technique can be implemented using, e.g., special instruments or designed software. Averaging process provide to increase in the *SNR* according to:

(13) $$\frac{S_{c}}{N_{c}}=S_{\text{ac}}{\left(\frac{N_{\text{ac}}}{\sqrt{n_{\text{smpl}}}}\right)}^{-1}$$

where *n_smpl_* is the number of the averaging samples. In the designed CEAS setup, these procedures makes it possible to reach a value of decay time *τ* with a determination uncertainty below 0.5% (e.g., in the case of 10,000 averaging samples).

## 4. Selected Experimental Applications

Many different technologies are applied to gas detection and identification. However, optoelectronic methods enable a direct and selective measurement of concentration at the level of a single ppb or even sub-ppb. It is noticed that they require common primary elements: the radiation source, sample gas cell, detection module, optics, readout electronics and software. These elements are selected taking into consideration principles of sensors operations. This section reviews some systems designed to detect nitrogen oxides. One of the most common gas detection systems is differential optical absorption spectroscopy. The first DOAS system was applied by Ulrich Platt in the 1970s. Currently, similar arrangements are applied to the monitoring of atmospheric pollutants, including the detection of NO_x_, in terrestrial applications, in the air and in the space, e.g., the GOME and SCIAMACHY satellites. Sensitivity of the method depends on the distance between the radiation source and the photoreceiver. For systems where this distance is a few kilometres, the obtained sensitivity is better than 1 ppb in the case of NO_2_ detection [74]. For NO detection, the absorption effect at the wavelength of 227 nm is used, but influence of other gases (NO_2_, SO_2_) at the same absorption line is observed. Their interferences are approximated analysing absorption effect at their specific spectral lines: NO_2_—413 nm, SO_2_—287 nm. The main elements of this system are a gas cell (with length of 1 m), a light source—(deuterium-halogen Lamp), and a detection module consisting of a spectrometer. Using this setup, a NO detection threshold of 5 ppm with a response time of 3.4 s was achieved [8].

**Figure 12.**
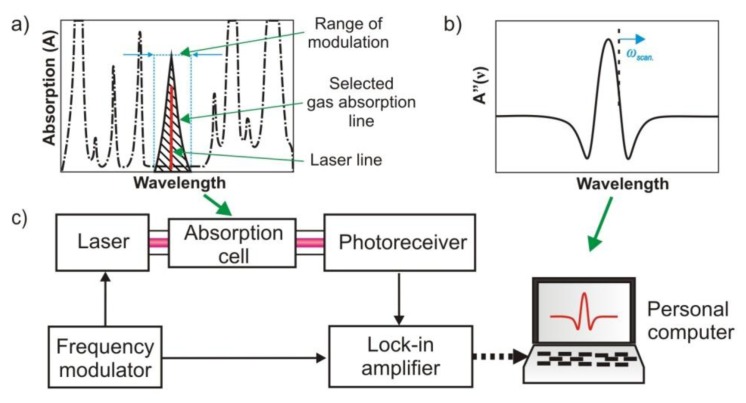
Operation idea of TLAS: spectrum scanning (**a**); the result of measurement (**b**); and experimental scheme (**c**).

In order to improve the sensitivity of absorption methods, reflective multipass cells are used, e.g., in tuneable laser absorption spectroscopy (Figure 12) [79]. Applications of cells with lengths of a few dozen meters provide the possibility to achieve a sensitivity of 1 ppb and higher [74]. In the TLAS gas absorption lines are analysed by optical radiation at a well-defined but adjustable or tuneable wavelength. The tuning mechanism depends on the type of the laser. Typically, rapid and precise tuning of the laser spectrum around the specified wavelength is obtained by special driving of the laser injection current and temperature settings. However, in the case of EC-QCL, some other tuning methods (rotation of the internal diffraction grating) are also available [12].

In detection systems using spectrum tuning, the changes of the signal are caused by fluctuation of laser power follows the wavelength modulation and target gas absorption. The modulation and lock-in detection technique is called wavelength modulation spectroscopy (WMS). WMS provides fast, sensitive, accurate, and *in situ* measurements for a wide variety of gas conditions, even in harsh, real-world environments [12]. In order to improve TLAS detection features such as speed and detection limits, high frequency modulation (FMS) techniques have been introduced. These techniques determine the absorption or dispersion of a narrow spectral feature by detecting the heterodyne beat signal that appears when the optical radiation is distorted by the spectral feature of interest. In comparison to the WMS, there is applied high frequency modulation instead of conventionally used kHz frequencies. At MHz to GHz frequencies, laser excess noise has been minimised and better detection limits can be obtained.

Summarizing, TLAS offers advantages over conventional spectrophotometry. It provides opportunity to determine amount of substance fractions of gases from major to trace levels. A detection limit of 10^−4^−10^−6^ cm^−1^ is obtained. Higher detection limits can be available with the use of other techniques, like CRDS, CEAS or QEPAS. Operation principles of CRDS and CEAS systems are described in the Introduction and in our research activities. Cavity leak-out spectroscopy is a cw variant of the CRDS [17]. After optical excitation of the cavity, the laser beam is turned off and the subsequent power decay of the radiation is registered by a photodetector. For example, the CALOS setup comprises a CO gas laser, a CdTe electro-optical modulator (EOM), a high-finesse ring-down cavity and a photodetector (a LN_2_ cooled InSb photodetector). For the generation of tunable sidebands, a laser beam is sent to the EOM, operating at microwave frequencies. This beam is periodically injected into the ring-down cell, twice per modulation period. Each time the transmitted light indicates optimum coincidence of laser frequency and cavity mode, a trigger pulse is provided to turn off the laser sideband radiation via the EOM. The decay time of each leak-out signal is determined by means of a fast exponential fitting algorithm. In the setup operated at the wavelength of 5.33 μm, a detection limit for NO in the ppt range was obtained [17].

Incoherent broadband cavity enhanced absorption spectroscopy is designed for detection of atmospheric trace gases. Practical realizations are similar to CEAS setups, however an incoherent light source (LED) is applied. This technique does not need mode matching, dithering the optical cavity or off-axis alignment. Additionally, a wide spectral range makes it possible to simultaneously detect multiple absorbers. The main disadvantage of the IBBCEAS is worse response time (the minutes time scale). For example, an IBBCEAS experimental setup can be built of a light source (high-power blue LED-450 nm with temperature stabilization), optical cavity with two mirrors characterized by high reflectivity of 99.7% around 480 nm and separated by a distance of 97.5 cm. The transmitted light from the cavity is analyzed using a high-resolution spectrometer (HR2000, Ocean Optics, Dunedin, FL, USA) in a Czerny-Turner configuration. For example, 18.1 ppbv NO_2_ in laboratory air was measured with a detection limit of about 2.2 ppbv using an optimal averaging time of 100 s [10].

**Figure 13.**
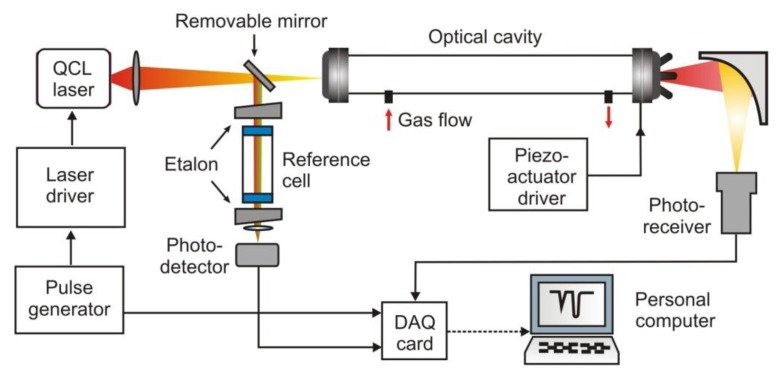
Operation principles of ICOS system.

The idea of integrated cavity output spectroscopy is similar like CEAS, however, the measuring procedure is based on comparison of the signal amplitudes at the input and the output of the cavity (Figure 13) [79,80].

In the case of off-axis illumination of the resonator, the wavelength and electric-field phase information can be neglected, leading to a simplified description of the output intensity:

(14) Iout=−Iin(1−R)2e−αL2ln(Re−αL)

where *I_out_*, *I_in_*—the values of signals at the output and input of the cavity, *R*—mirrors reflectances, *L*—distance between cavity mirrors.

While, the single pass output intensity can be calculated from:

(15) $$I^{'}=I_{\text{in}}{\left(1-R\right)}^{2}e^{-\alpha L}$$

Due to the energy storage in the cavity, the output intensity is larger than the intensity *I'* corresponding to situation when the radiation is not trapped in the resonator. One can show that for ICOS method, very useful expression is:

(16) $$\alpha =\frac{\ln(R)}{L}\frac{I-I^{'}}{I}$$

A disadvantage of the ICOS approach consists in the fact that precise information about mirror reflectivity is required. Additionally, the laser pulses are not locked on each cavity mode but sweeping gas absorption line. Laser beam provides spatially separated, multiple reflections. Therefore, the ICOS is effectively lowering the cavity free spectral range, producing a dense mode structure. In such an alignment, many cavity modes exist and the laser linewidth should be broad, but narrow with regard to the molecular line. In comparison with the CRDS, there is no limitation concerning ring-down time or mode matching between the laser frequency and the cavity free spectral range.

Faraday modulation spectrometry is another technique that provides an increase in the detection limit of direct laser absorption spectroscopy by 2–3 orders of magnitude [9,12]. A magnetic field, which breaks the magnetic degeneracy of the rotational states, is applied. It is the so called Zeeman Effect that causes a frequency shift of transitions. The shift is different for left-handed and right-handed circularly polarized optical radiation. The polarization state of incident linearly polarized light in the presence of a magnetic field can be changed by a transition in a paramagnetic molecule. The presence of an absorber is detected by the level of signal (light absorption), conversion of polarization state (linear-elliptical) and rotation of polarization plane. Since the polarization rotation exists only when the molecules of the absorber are present, ultra-high sensitivities are obtained with this method. The detection system is built of a light source, two nearly crossed Rochon polarizers, long optical cell, sensitive detector, lock-in amplifier, a magnetic field unit with power amplifier (a copper wire coil inside the cell). There have been presented two FRS detection systems operated at two different wavelength ranges [9,12]. The first one uses a fully-diode-laser-based UV laser system and GaP UV-enhanced photodiode. In the second setup, a tunable external cavity quantum cascade laser and liquid nitrogen-cooled indium-antimonide photodetector were applied. These setups are characterized by the minimum detectable concentration of NO at the level of 3.4 ppb (UV—227 nm) and 380 ppt (IR—5.33 μm).

**Figure 14.**
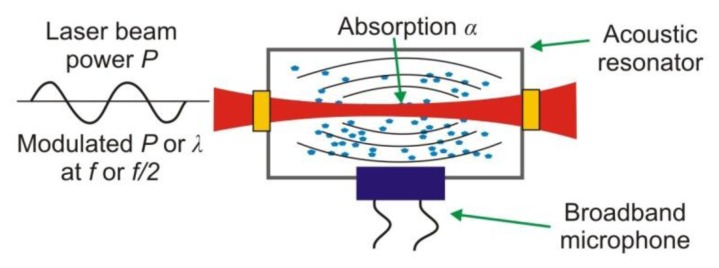
Idea of photoacoustic spectroscopy.

In the photoacoustic spectroscopy (PAS), the conversion of light to sound in absorbing materials is used [12]. The photoacoustic signal is traditionally detected using a resonant acoustic cell equipped with a sensitive microphone (Figure 14) [79].

The signal shape registered by microphone depends on the absorber concentration in the investigated sample. The main disadvantage of PAS sensors is sensitivity to mechanical and acoustic vibrations. In order to increase in the immunity for these noises various acoustic resonators are applied. Effectiveness of these solutions is limited when the conventional microphones of a flat frequency response are used.

**Figure 15.**
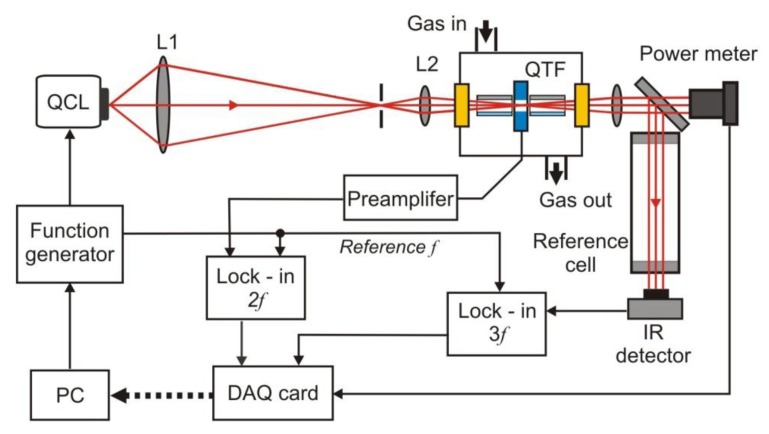
Setup of quartz-enhanced photoacoustic spectroscopy system.

The sensitivity of the PAS was recently increased many times due to the use of resonance quartz forks (QTFs) [12,18]. Such technique is called quartz-enhanced photoacoustic spectroscopy (QEPAS). The QEPAS is characterized by a simple design (Figure 15), immunity to environmental acoustic noise, applicability over a wide range of pressures, and the capability to analyze small gas samples, down to 1 mm^3^ in volume. Low-frequency quartz tuning fork (QTF’s) with the resonance at 32.8 kHz in vacuum are applied [12]. Only the symmetric vibration of a QTF is piezoelectrically active. The excitation beam passes through the gap between the QTF prongs for efficient excitation of this vibration.

Acoustically, a QTF is a quadrupole, which results in excellent environmental noise immunity. Sound waves from acoustic sources tend to move the QTF prongs in the same direction, thus resulting in no electrical response. The QTF deformation results in the generation of electrical charges on its electrode pairs. In the setup, measurements are usually made with a wavelength modulation technique and *2f* detection method, which suppresses the background originating from spectrally non-selective absorbers (such as resonator walls, QTF electrodes, and the gas cell elements). As a radiation source, EC-QCL is usually applied. A lock-in amplifier is used to demodulate the QTF signal. Spectral data can be acquired if the laser wavelength is tuned. To increase the effective interaction length between the radiation-induced sound and the QTF, an acoustic gas-filled resonator can be added similarly to the traditional PAS approach. In addition reference cell and IR detector (often pyro detector) to registration *1f* or *3f* signal is applied. The QEPAS provides opportunity to construct the sensors of the best detection sensitivity [18].

## 5. Review of Our Applications

This section concerns the review of our cavity enhanced systems designed for nitrogen oxides detection. They operate in the visible and mid-infrared wavelengths based on cavity enhanced absorption spectroscopy. Each of the sensors is characterized by different performances, but the main aspects concern the development of unique multi-channel detection systems.

**Figure 16.**
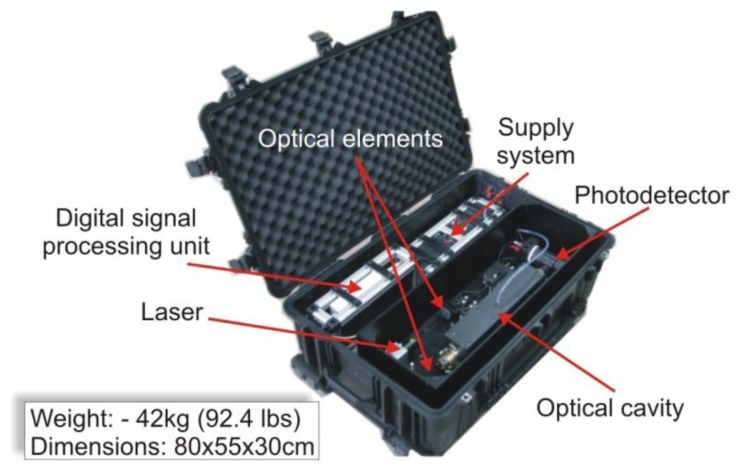
Portable NO_2_ sensor.

One of the our instrument is the *VIS-sensor* designed for nitrogen dioxide detection. The sensor consists of a pulsed laser diode, diffraction grating and mirror, optical cavity, detection module with photomultiplier tube (PMT), digital signal processing unit with special software (Figure 16).

The laser generates radiation pulses at the wavelength of 414 nm and with a 1 kHz repetition rate. The light pulses are directed to the cavity using the diffraction grating and external mirror. The optical cavity is built with two mirrors, the reflectivities of which reach a value of 0.99995 at the wavelength of interest. The distance between the cavity mirrors is 60 cm. The leakage radiation from the cavity is registered with a detection module consisting of a photomultiplier tube (R7518, Hamamatsu, Iwata, Japan) and preamp (transimpedance). The output signal from the module is digitized with a fast 14-bit data acquisition card (CS328, Cleverscope, Epsom, New Zealand). In the sensor are also applied a system of coalescence filters and a special heating unit for the mirrors. The filtering minimizes light scattering caused by some aerosols and smokes existing in the air. While the mirrors heating to the temperature of 50 °C prevents water vapour condensation on their surface. Its portable construction as well as low energy consumption provides the opportunity to use it in different applications.

**Figure 17.**
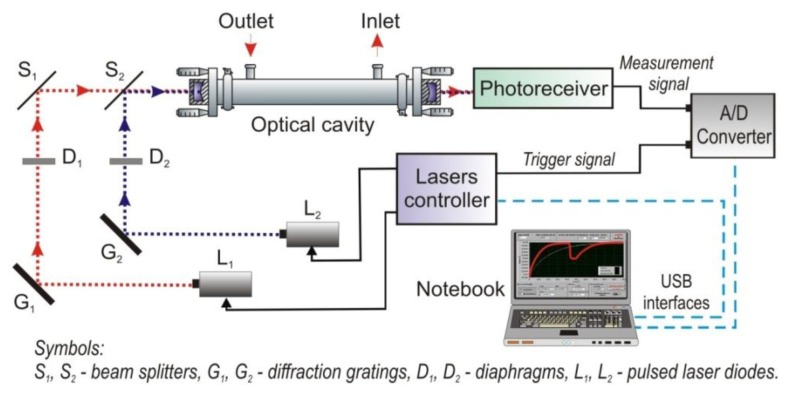
Scheme of two-spectral CEAS sensor.

The second instrument is a VIS-two-channel sensor. Figure 17 shows an experimental CEAS setup using two detection channels with the same optical cavity and one photoreceiver [81]. The setup makes it possible to detect a trace concentration of two gases with a different absorption spectrum at the same time. Another possibility is applying one of the channels for alignment control of the optical elements. Therefore, a special signal processing unit for registration of two optical signals was developed. In the unit, technique of time domain multiplexing of lasers was applied. Each of the lasers is assigned to the suitable measurement channel. The leakage radiation for two laser beams is detected in the strictly defined temporal window. The decay time *τ_0_* for the cavity without absorber is the main parameter determined duration of the temporal window.

As a radiation source two laser diodes were applied. The lasers are designed for cw mode operating at room temperatures. In the first channel, the AlGaInP laser diode (635 nm) was used (type DL-5038-031, SANYO Electric Co., Ltd., Moriguchi-shi, Japan). The second channel is equipped with a violet laser diode (410 nm) type GH04125A2A (Sharp Microelectronics of the Americas, Camas, WA, USA). Next, the laser beams were shaped with diffraction gratings and diaphragms, and were directed with beam splitters (CVI Melles Griot, Rochester, NY, USA) into the optical cavity. The optical cavity was built with two spherical mirrors with a reflectivity of about 0.999 at the wavelengths of interest. The distance between the mirrors was 50 cm. For detection of leakage radiation, the photomultiplier R7518 type from Hamamatsu was used. The construction of both detection channels and signal processing algorithm are similar to the NO_2_ sensor described in Section 4.1.

**Figure 18.**
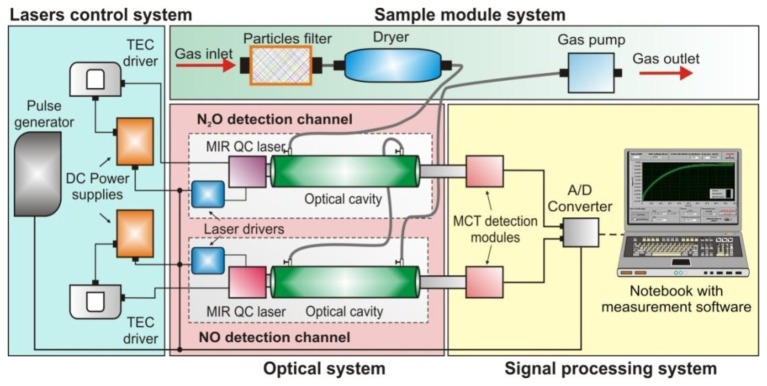
Block diagram of the NO and N_2_O sensor.

We also developed some sensors operating in the mid-infrared wavelength range. An example of such sensors is a MIR-two-channel sensor designed for NO and N_2_O detection. The project of the NO and N_2_O sensor is presented in Figure 18.

It is built with a laser control system, optical system, sample module and signal processing unit. In the sensor, two quantum cascade lasers were applied. Their emission lines are very narrow and also are characterised by both high power and good spectral stability. The high sensitivity of the sensor is obtained by matching wavelengths of the QC lasers radiation to the selected absorption lines of the tested gases: 5.27 μm (for NO) and 4.53 μm (for N_2_O). The optical cavity was built with two high reflective mirrors (*R* > 0.995) located with the distance of about 60 cm. The leakage radiation from the cavities was registered with two optimized detection modules—PVI-2TE (VIGO System S.A., Ożarów Mazowiecki, Poland). The main elements of the modules are MCT photodetectors, transimpedance preamplifiers and TEC units [72,82]. Next, signals from the preamplifiers were digitized using an A/D converter.

**Figure 19.**
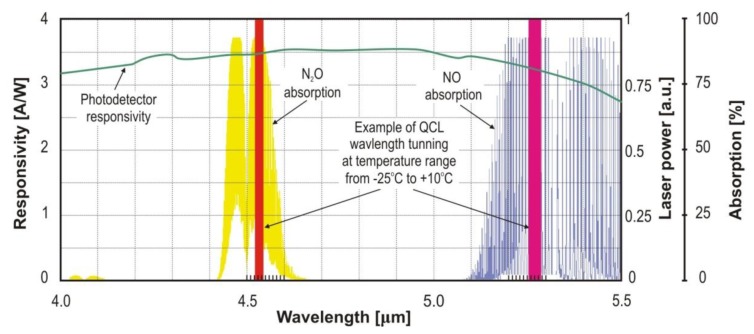
Characteristics of the selected QC laser spectra, N_2_O and NO absorption lines and photodetector responsivity.

In Figure 19, the relations between tuning spectral ranges of selected QC lasers, photodetector responsivity, and selected N_2_O and NO absorption spectra are shown.

In our sensors, determination of tested gas concentrations is based on measurements of the optical radiation changes. The changes are caused by the light absorption phenomenon. The measurement was carried out during a two-step process. First, the decay time *τ_0_* of lasers radiation in each optical cavity without an absorber was found. It provided an opportunity to determine the *Q*-factors for ‘clear’ conditions. Then, the cavities were filled with the absorbing mixture and the respective decay time values *τ_1_* were measured. Knowing the absorption cross section *σ* of the compounds, their concentrations in respective cavities were calculated from the formula:

(18) $$N_{x}=\frac{1}{N_{o}c\sigma }\left(\frac{1}{\tau_{1}}-\frac{1}{\tau_{0}}\right)$$

where *N_o_* denotes the Loschmidt’s number, while *c* is the light speed.

**Figure 20.**
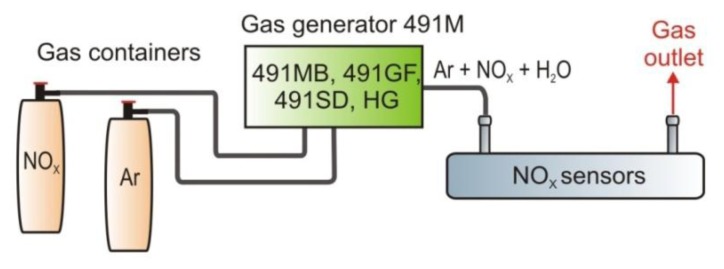
Block diagram of the setup designed for the sensor testing.

During the sensor investigation, concentration measurements of reference gas samples were carried out. Gas samples were prepared using the 491M type gas standards generator from KIN-TEK Laboratories, Inc. (La Marque, TX, USA, Figure 20).

The modular construction of the instruments enables production of gas mixtures. The high precision of the generator from a level of part per trillion (ppt) to the initial concentration of 1:1 is guaranteed. The instrument also produces both dry and moistened standard gas, which can be supplied to the sensor at an adjustable pressure.

**Figure 21.**
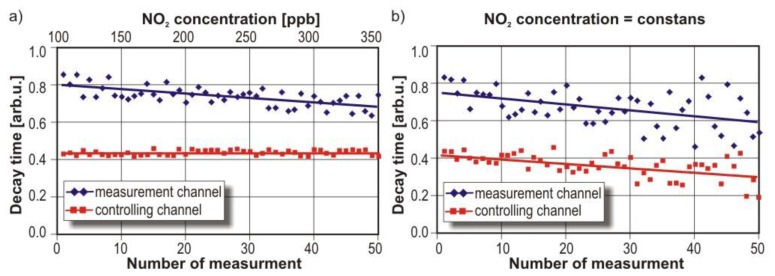
Example of results of different NO_2_ concentrations measurements (**a**); concentration measurements of humid mixture of NO_2_ (**b**).

Each of the presented sensors was tested. Example results are discussed in the case of the *VIS-two-channel* sensor. Experiments were performed using one channel of the sensor for nitrogen dioxide concentration measurements and the second channel for cavity quality control. The control process was performed continuously by using the second channel outside the absorption spectrum of NO_2_. Measurements were carried out for the cavity with the different NO_2_ concentrations (from 100 ppb–345 ppb) and with humid mixture of nitrogen dioxide. The relative humidity of the gas samples was 80%. Example of research results are presented in Figure 21.

In the case of the cavity with dry mixtures of different NO_2_ concentration, the average decay time in the control channel and measurement results channel were constant (Figure 20(a)). While in the second channel the decay time decreased proportionally to the NO_2_ concentration. When the humid mixture of NO_2_ was delivered to the sensor, both decay times were decreased (Figure 20(b)). It was caused by moisture, which distorted cavity mirrors reflectance.

Thus, the reason of the signal decreasing in the NO_2_ channel can be identified as a moisture influence. If nitrogen dioxide concentration is increased, only the decay time measured in the channel with a violet laser is decreased.

In Table 4, the results of the tests for different types of developed sensors are listed. Presented systems are characterized by different parameters. These are related to different absorption cross sections of the detected molecules, to different spectral ranges (others lasers, photodetectors and optical components). Moreover, in the systems using optical cavities contain mirrors which reflectivity is different. Sensor parameters like detection limit and uncertainty are described more detailed in [73].

## 6. Examples of Explosive Detection Applications

The designed detection systems can be applied to air quality control or in human breath analysis [79]. Moreover, the detection of vapours of some explosives is also possible. Some successful research with nitroglycerine (NG), nitrocellulose and TNT has been already performed [83].

**Figure 22.**
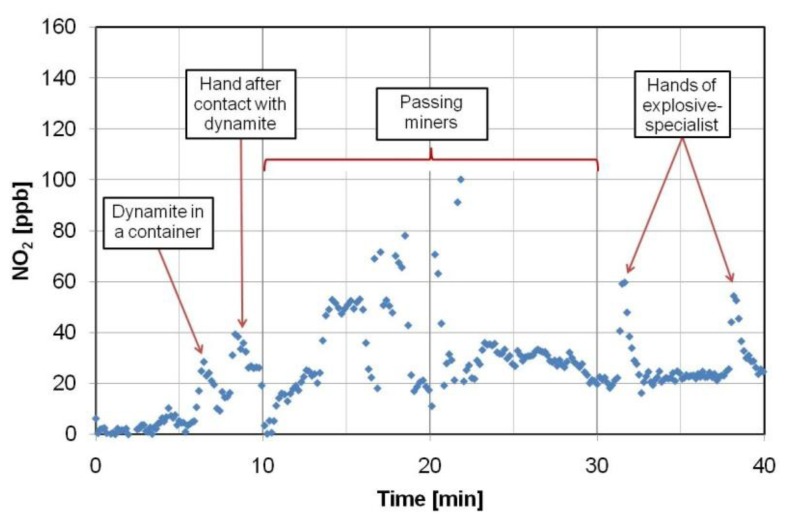
Detection of explosives with NO_2_ optoelectronic sensor in Polish mine [84].

Application tests of the portable NO_2_ optoelectronic sensor were performed in a Polish mine, where explosive devices with NG (dynamite) and ammonium nitrate were used. The measurements were carried out in specific and harsh conditions at a ground depth about 1,100 m. Several investigation scenarios were prepared. During the first step of the experiment, the explosives were searched for in various containers. For example, the peak detection signal (at least 20 ppb of NO_2_ above background level) was observed during examination of a non-hermetic package that contained dynamite (Figure 22).

Other investigations concerned people, their clothes and equipment. The detection peaks were recorded during monitoring of workers’ hands that had touched the explosives and objects with traces of NG (even 40 ppb of NO_2_ above background level) [84].

The experiments showed that the NO_2_ sensor enables differentiating people and objects that came into contact with the explosives characterized by high pressure of the vapours as well as to find the explosives in a non-hermetic package.

For detection of explosives with lower pressure of the vapours, a special concentrator system was also developed [83,84]. In the system, an adsorbent composed of Al_2_O_3_ (95%) and CeO_2_ (5%) as well as commercial adsorbents Tenax and Carboxen were applied. This system makes it possible to collect particles of explosives and to perform their thermal decomposition. Thanks to this, an increase in nitrogen oxide concentrations was possible, so during the next experiment the detection of nitrogen oxides produced during thermal decomposition of explosives was carried out. Thermal decomposition of explosives was carried out in an argon atmosphere and at different values of sorbent temperatures. The measurement showed that the highest concentrations were recorded using Carboxen. The concentration of nitrous oxide varied in the range from tens to hundreds of ppb for all 1 ng samples of explosives. In the same setup, the concentration of nitric oxide reached much higher values (ppm range). For TNT and PETN a significant increase in NO_x_ concentration was observed at the temperature of 160 °C. In comparison, for RDX and HMX the growth in concentration of nitrogen oxides was observed at higher temperatures. It can be explained by different values of their vapour pressure. Hexogen and octogen are characterized by a lower vapour pressure than TNT and PETN. Experiments showed that the sensor was able to detect the explosives portions of 1 ng such as TNT, PETN, RDX and HMX.

In comparison, there are some other optical methods of explosives detection [83]. For example, explosives sensing can be obtained using surface enhanced Raman scattering (SERS) [85], laser-induced fluorescence (LIF) [86], laser induced break-down spectroscopy (LIBS) [87] or by use of terahertz radiation [88]. Application of absorption spectroscopy to explosive detection is used as well. There are examples of military applications of LIDAR. As were described in literatures the standoff optical methods deliver the sensitivities reaching even the ppb level [89,90]. Methods based on the photoacoustic effect and in multipass spectroscopy were also used to explosive detection. For example, there was reported application PAS system to TNT spectrum investigation. The detection limit corresponds to saturated TNT vapour pressure at 5 °C was reached, *i.e.*, about 0.1 ppb [12]. As a radiation source EC-QCL with radiation wavelength near 7.3 μm was used.

## 7. Conclusions

In the paper, a description of the cavity enhanced absorption spectroscopy and its application to nitrogen oxide detection in the visible and mid-infrared range of wavelengths are presented. The selection of the detection spectral range, photoreceiver *SNR* analyses, and a review of commercial lasers and photodetectors available on the market were briefly described. We also presented some developed optoelectronic sensors designed to detect some nitrogen oxides. They are designed for *in-situ* gas concentrations measurements. We have also shown that our NO_x_ sensors can detect nanogram levels of explosives like TNT, PETN, RDX and HMX.

Our further research concerns the application of such sensors in human breath analysis. It provides the possibility to detect pathogenic changes at the molecular level. The presence of certain molecules in the exhaled air will be used as an indicator of specific diseases.

## Figures and Tables

**Table 1.** Detection limit of technique examples used for NO_x_ detection.

**Detection Method**	**Type of Gas**	**Range of Wavelengths**	**Detection Limit**

Differential Optical Absorption Spectroscopy [8]	NO	UV	5 ppm
Faraday Modulation Spectroscopy (FAMOS) [9]	NO	UV	3.4 ppb
Incoherent Broadband Cavity Enhanced Absorption Spectroscopy (IBBCEAS) [10]	NO_2_	UV	2.2 ppb
Tunable Laser Absorption Spectroscopy (TLAS) [11]	NO_2_	VIS	5 ppb
Faraday Rotation Spectrometer (FRS) [12]	NO	IR	380 pptv
Cavity Enhanced Absorption Spectroscopy [13]	NO	IR	16 ppb
Cavity Ring-Down Spectroscopy [14]	NO	IR	0.7 ppb
Wavelength Modulation Spectroscopy (WMS) [15]	NO	IR	0.3 ppm
Tunable Laser Absorption Spectroscopy [16]	NO	IR	2 ppb
Cavity Leak-Out Spectroscopy (CALOS) [17]	^14^NO, ^15^NO	IR	7 ppt
Quartz Enhanced Photoacoustic Spectroscopy (QEPAS) [18]	NO	IR	5 ppbv
Integrated Cavity Output Spectroscopy [19]	NO_2_	IR	26 ppt

**Table 2.** Example parameters of selected IR lasers.

**Laser Type/Manufacturer**	**Power** **(W)**	**Wavelength** **(μm)**	**Operation** **Temperature (K)**	**Spectral** **Tuning (cm^-1^)**
DFB-QCL [62]	2.4	4.8	RT	4.0
RCSE-QCL [63]	0.4	4.9	RT	-
QCL array [64]	1.1	9	RT	220
Multi-section QCL [65]	0.08	9.3	RT	450
EC-QCL [66]	0.035	3.2	RT	83
Optically tunable QCL [67]	0.10	9.0	RT	0.3

**Table 3.** Example parameters of selected detectors.

**Type of Photodetector** **(Manufacturer)**	**Active Area** **Dimensions** **(mm)**	Detectivity D* (cmHz^1/2^W^-1^)	**Operating Temperature** ***(LN_2_—Temperature of Liquid Nitrogen*,** ***RT—Room Temperature)***
InSb (JUDSON)	0.1–7.0	10^11^	LN_2_
PbSe (JUDSON)	1–3	5 × 10^9^–2 × 10^10^	RT
HgCdTe PC (JUDSON)	0.05–4.00	4 × 10^9^–1 × 10^11^	LN_2_/RT
HgCdTe PV (JUDSON)	0.25–1.00	4 × 10^9^–1.1 × 10^12^	RT
InSb (InfraRed)	0.25–2.00	>10^11^	LN_2_
HgCdTe (InfraRed)	0.1–2.0	10^10^–4×10^10^	RT
HgCdTe (VIGO System)	0.025–4.0	>10^11^	RT
QWIP GaAs/GaAlAs [72]	-	10^10^	LN_2_/RT
SL InAs-GaSb [72]	-	10^11^	RT

**Table 4.** The test results of our sensors.

**Type of Sensor**	**Operation Wavelength**	**Detected Gas**	**Detection Limit**	**Measurement Uncertainty**
VIS-one-channel	414 nm	NO_2_	1 ppb	5%
VIS-two-channels	410 nm 635 nm	NO_2_ control channel	30 ppb -	3% -
MIR-two-channels	5.27 μm 4.53 μm	NO N_2_O	70 ppb 45 ppb	12% 13%

## References

[1] Moore D.S. (2007). Recent advances in trace explosives detection instrumentation. Sens. Imaging.

[2] Roller C., Namjou K., Jeffers J.D., Camp M., Mock A., McCann P.J., Grego J. (2002). Nitric oxide breath testing by tunable-diode laser absorption spectroscopy: Application in monitoring respiratory inflammation. Appl. Opt..

[3] Sigrist M.W. (1994). Air Monitoring by Spectroscopic Techniques.

[4] Li P., Shi K., Liu Z. (2005). Optical scattering spectroscopy by using tightly focused supercontinuum. Opt. Express.

[5] Lagalante A.F. (1999). Atomic absorption spectroscopy: A tutorial review. Appl. Spectrosc. Rev..

[6] O’Keefe A. (1998). Integrated cavity output analysis of ultra-weak absorption. Chem. Phys. Lett..

[7] Noel S., Bovensmann H., Burrows J.P., Frerick J., Chance K.V., Goede A.H.P. (1999). Global atmospheric monitoring with SCIAMACHY. Phys. Chem. Earth.

[8] Dooly G., Fitzpatrick C., Lewis E. (2008). Deep UV based DOAS system for the monitoring of nitric oxide using ratiometric separation techniques. Sens. Actuators B.

[9] Shao J., Wang H., Zhou W., Peng B., Ying C. The Highly Sensitive Detection of NO Using FAMOS by a Fully-Diode-Laser-Based UV System.

[10] Wu T., Zhao W., Chen W., Zhang W., Gao X. (2009). Incoherent broadband cavity enhanced absorption spectroscopyfor in situ measurements of NO_2_ with a blue light emitting diode. Appl. Phys. B.

[11] Sonnenfroh D.M., Allen M.G. (1996). Ultrasensitive, visible tunable diode laser detection of NO_2_. Appl. Opt..

[12] Curl R.F., Capasso F., Gmachl C., Kosterev A.A., McManus B., Lewicki R., Pusharsky M., Wysocki G., Tittel F.K. (2010). Quantum cascade lasers in chemical physics. Chem. Phys. Lett..

[13] Menzel L., Kosterev A.A., Curl R.F., Tittel F.K., Gmachl C., Capasso F., Sivco D.L., Baillargeon J.N., Hutchinson A.L., Cho A.Y. (2001). Spectroscopic detection of biological NO with a quantum cascade laser. Appl. Phys. B.

[14] Kosterev A.A., Malinovsky A.L., Tittel F.K., Gmachl C., Capasso F., Sivco D.L., Baillargeon J.N., Hutchinson A.L., Cho A.Y. (2001). Cavity ringdown spectroscopic detection of nitric oxide with a continuous-wave quantum-cascade laser. Appl. Opt..

[15] Chao X., Jeffries J.B., Hanson R.K. (2012). Wavelength-modulation-spectroscopy for real-time, *in situ* NO detection in combustion gases with a 5.2 μm quantum-cascade laser. Appl. Phys. B.

[16] Namjou K., Roller C.B., McMillen G. Breath Analysis Using Mid Infrared Tunable Laser Spectroscopy.

[17] Heinrich K., Fritsch T., Hering P., Mürtz M. (2009). Infrared laser-spectroscopic analysis of ^14^NO and ^15^NO in human breath. Appl. Phys. B.

[18] Tittel F.K., Dong L., Lewicki R., Lee G., Peralta A., Spagnolo V. (2012). Sensitive detection of nitric oxide using 5.26 μm external cavity quantum cascade laser based QEPAS sensor. Proc. SPIE.

[19] Karpf A., Rao G.N. High Sensitivity Detection of NO_2_Using ICOS and MLIAS.

[20] Busch K.W., Busch M.A. (1999). Cavity-Ringdown Spectroscopy.

[21] Engeln R., Berden G., Peeters R., Meijer G. (1998). Cavity enhanced absorption and cavity enhanced magnetic rotation spectroscopy. Rev. Sci. Instrum..

[22] Wojtas J., Czyżewski A., Stacewicz T., Bielecki Z., Mikolajczyk J. (2005). Cavity enhanced spectroscopy for NO_2_detection. Proc. SPIE.

[23] Paul J.B., Lapson L., Anderson J.G. (2001). Ultrasensitive absorption spectroscopy with a high-finesse optical cavity and off-axis alignment. App. Opt..

[24] Berden G., Peeters R., Meijer G. (2000). Cavity ring-down spectroscopy: Experimental schemes and applications. Int. Rev. Phys. Chem..

[25] Courtillot I., Morville J., Motto-Ros V., Romanini D. (2006). Sub-PPB NO_2_ detection by optical feedback cavity-enhanced absorption spectroscopy with a blue diode laser. Appl. Phys. B.

[26] Wojtas J., Czyzewski A., Stacewicz T., Bielecki Z. (2006). Sensitive detection of NO_2_ with cavity enhanced spectroscopy. Opt. Appl..

[27] Nowakowski M., Wojtas J., Bielecki Z., Mikolajczyk J. (2009). Cavity enhanced absorption spectroscopy sensor. Acta Phys. Pol. A.

[28] Stacewicz T., Wojtas J., Bielecki Z., Nowakowski M., Mikołajczyk J., Mędrzycki R., Rutecka B. (2012). Cavity ring down spectroscopy: Detection of trace amounts of matter. Opt. Electron. Rev..

[29] High-Resolution Transmission Molecular Absorption Database—HITRAN (2008). http://www.hitran.com.

[30] Rutecka B., Wojtas J., Bielecki Z., Mikolajczyk J., Nowakowski M. (2010). Application of an optical parametric generator to cavity enhanced experiment. Proc. SPIE.

[31] Commercial Lasers Lines http://photonics.com/LinearCharts/Default.aspx?ChartID=1.

[32] TopGaN Ltd http://topganlasers.com/.

[33] Roithner Lasertechnik GmbH http://www.roithner-laser.com/.

[34] UV, Visible, & IR Laser Diodes http://www.powertechnology.com/diodes.asp.

[35] QPhotonics: Laser Diode Online Store http://www.qphotonics.com/home.php?xid=2661dc5f744a077a186530c93bb4888c.

[36] De Natale P., Ferraro P. (2006). Advanced monitoring techniques and coherent sources. Opt. Lasers Eng..

[37] Nilsson J., Jeong Y., Soh D.B.S., Codemard C.A., Dupriez P., Farell C., Sahu J.K., Kim J., Yoo S., Payne D.N. High-Power Fiber Lasers: Progress and Opportunities.

[38] Zybin A., Koch J., Wizemann H.D., Franzke J., Niemax K. (2005). Diode laser atomic absorption spectrometry. Spectrochim. Acta B.

[39] Kasyutich V.L., Canosa-Mas C.E., Pfrang C., Vaughan S., Wayne R.P. (2002). Off-axis continuous-wave cavity-enhanced absorption spectroscopy of narrow-band and broadband absorbers using red diode lasers. Appl. Phys. B.

[40] Kebabian P.L., Herdon S.C., Freedman A. (2005). Detection of nitrogen dioxide by cavity attenuated phase shift spectroscopy. Anal. Chem..

[41] Kebabian P.L., Wood E.C., Herdon S.C., Freedman A. (2008). A practical alternative to chemiluminescence-based detection of nitrogen-dioxide: Cavity attenuated phase shift spectroscopy. Environ. Sci. Tech..

[42] Osthoff H.D., Brown S.S., Ryerson T.B., Fortin T.J., Lerner B.M., Williams E.J., Pettersson A., Baynard T., Dubé W.P., Ciciora S.J. (2006). Measurement of atmospheric NO_2_ by pulsed cavity ring-down spectroscopy. J. Geophys. Res..

[43] Tittel F.K., Richter D., Fried A., Sorokina I.T., Vodopyanov K.L. (2003). Mid-Infrared Laser Applications in Spectroscopy. Solid-State Mid-Infrared Laser Sources.

[44] Kosterev A., Wysocki G., Bakhirkin Y., So S., Lewicki R., Fraser M., Tittel F.K., Curl R.F. (2008). Application of quantum cascade lasers to trace gas analysis. Appl. Phys. B.

[45] Yao Y., Wang X., Fan J.-Y., Gmachl C.F. (2010). High performance continuum-to-continuum quantum cascade lasers with a broad gain bandwidth of over 400 cm^‣1^. Appl. Phys. Lett..

[46] Zhao L., Liu F., Zhang J., Wang L., Liu J., Li L., Wang Z. (2012). Improved performance of quantum cascade laser with porous waveguide structure. J. Appl. Phys..

[47] Liu P.Q., Wang X., Fan J.-Y., Gmachl C.F. (2011). Single-mode quantum cascade lasers based on a folded Fabry-Perot cavity. Appl. Phys. Lett..

[48] Bai Y., Tsao S., Bandyopadhyay N., Slivken S., Lu Q.Y., Caffey D., Pushkarsky M., Day T., Razeghi M. (2011). High power, continuous wave, quantum cascade ring laser. Appl. Phys. Lett..

[49] Wang L.J., Zhai S.Q., Yao D.Y., Liu J.Q., Wang Z.G., Zhang J.C., Liu F.Q. (2013). High temperature operation of edge-emitting photonic-crystal distributed-feedback quantum cascade lasers at λ~7.6 μm. Phys. E.

[50] Tsai T., Wysocki G. (2010). External-cavity quantum cascade lasers with fast wavelength scanning. Appl. Phys. B.

[51] Kasyutich V.L., Raj I.R.K., Martin P.A. (2010). Stability of widely tuneable, continuous wave external-cavity quantum cascade laser for absorption spectroscopy. Infrared Phys. Technol..

[52] Liu P.Q., Wang X., Gmachl C.F. (2012). Single-mode quantum cascade lasers employing asymmetric Mach-Zehnder interferometer type cavities. Appl. Phys. Lett..

[53] Fuchs P., Seufert J., Koeth J., Semmel J., Höfling S., Worschech L., Forchel A. (2010). Widely tunable quantum cascade lasers with coupled cavities for gas detection. Appl. Phys. Lett..

[54] Daylight Solutions’ Products Based on ECacL(TM) Technology http://www.daylightsolutions.com/products/.

[55] Maulini R. (2006). Broadly Tunable Mid-Infrared Quantum Cascade Lasers for Spectroscopic Applications. Ph.D. Thesis.

[56] Straub A., Gmachl C., Sivco D.L., Sergent A.M., Capasso F., Cho A.Y. (2002). Simultaneously at two wavelengths (5.0 and 7.5 μm) singlemode and tunable quantum cascade distributed feedback lasers. Electron. Lett..

[57] Gmachl C., Sivco D.L., Colombelli R., Capasso F., Cho A.Y. (2002). Ultra-broadband semiconductor laser. Nature.

[58] Bandyopadhyay N., Slivken S., Bai Y., Razeghia M. (2012). High power, continuous wave, room temperature operation of λ~3.4μm and λ~3.55μm InP-based quantum cascade lasers. Appl. Phys. Lett..

[59] Laffaille P.J., Moreno C., Teissier R., Bahriz M., Baranov A.N. (2012). High temperature operation of short wavelength InAs-based quantum cascade lasers. AIP Adv..

[60] Bauer A., Langer F., Dallner M., Kamp M., Motyka M., Sek G., Ryczko K., Misiewicz J., Höfling S., Forchel A. (2009). Emission wavelength tuning of interband cascade lasers in the 3–4 μm spectral range. Appl. Phys. Lett..

[61] Basnar B., Mujagic E., Andrews A.M., Roch T., Schrenk W., Strasser G. (2010). Light induced tuning of quantum cascade lasers. Appl. Phys. Lett..

[62] Lu Q.Y., Bai Y., Bandyopadhyay N., Slivken S., Razeghi M. (2011). 2.4 W room temperature continuous wave operation of distributed feedback quantum cascade lasers. Appl. Phys. Lett..

[63] Mujagic E., Schwarzer C., Nobile M., Detz H., Ahn S., Schrenk W., Chen J., Gmachl C., Strasser G. Reduced Threshold and High Temperature Operation in Single-Mode Ring Cavity Surface Emitting Quantum Cascade Lasers.

[64] Lee B.G., Zhang H.A., Pflügl C., Diehl L., Belkin M.A., Fischer M., Wittmann A., Faist J., Capasso F. (2009). Broadband distributed-feedback quantum cascade laser array operating from 8.0 to 9.8 μm. IEEE Phot. Techn. Lett..

[65] Slivken S., Bandyopadhyay N., Tsao S., Nida S., Bai Y., Lu Q.Y., Razeghi M. (2012). Sampled grating, distributed feedback quantum cascade lasers with broad tunability and continuous operation at room temperature. Appl. Phys. Lett..

[66] Kruczek T., Fedorova K.A., Sokolovskii G.S., Teissier R., Baranov A.N., Rafailov E.U. (2013). InAs/AlSb widely tunable external cavity quantum cascade laser around 3.2 μm. Appl. Phys. Lett..

[67] Suchalkin S., Jung S., Tober R., Belkin M.A., Belenky G. (2013). Optically tunable long wavelength infrared quantum cascade laser operated at room temperature. Appl. Phys. Lett..

[68] Rogalski A., Bielecki Z. (2006). Detection of Optical Radiation. Handbook of Optoelectronics.

[69] Photonic Devices, Electron Tube Devices and Applied Products (2012). http://sales.hamamatsu.com/assets/pdf/catsandguides/p-dev_2012_TOTH0020E02.pdf.

[70] Hamamatsu, Solid State Division Technical Information SD-12, Characteristics and Use of Infrared Detectors. http://sales.hamamatsu.com/assets/applications/SSD/Characteristics_and_use_of_infrared_detectors.pdf.

[71] Piotrowski A., Madejczyk P., Gawron W., Klos K., Romanis M., Grudzien M., Rogalski A., Piotrowski J. (2004). MOCVD growth of Hg1xCdxTe heterostructures for uncooled infrared photodetectors. Opt. Electron. Rev..

[72] Rogalski A. (2012). History of infrared detectors. Opt. Electron. Rev..

[73] Wojtas J., Bielecki Z. (2008). Signal processing system in the cavity enhanced spectroscopy. Opt. Electron. Rev..

[74] Wojtas J. (2011). Detection of Optical Radiation in NO_x_Optoelectronic Sensors Employing Cavity Enhanced Absorption Spectroscopy. Optoelectronics—Devices and Applications.

[75] Flyckt S.O., Marmonier C. (2002). Photomultiplier Tubes: Principles and Applications.

[76] Bielecki Z. (2002). Maximization of signal to noise ratio in infrared radiation receivers. Opt. Electron. Rev..

[77] Bielecki Z., Kolosowski W., Sedek E., Wnuk M., Wojtas J. (2009). Multispectral Detection Circuits in Special Application. Transactions on Modelling and Simulations.

[78] Lyons R.G. (2010). Understanding Digital Signal Processing.

[79] Wojtas J., Bielecki Z., Stacewicz T., Mikolajczyk J., Nowakowski M. (2012). Ultrasensitive laser spectroscopy for breath analysis. Opt. Electron. Rev..

[80] Baer D.S., Paul J.B., Gupta M., O’Keefe A. (2002). Sensitive absorption measurements in the near-infrared region using off-axis integrated-cavity-output spectroscopy. Appl. Phys. B.

[81] Wojtas J. (2011). Two-channel optoelectronic sensor employing cavity enhanced absorption spectroscopy. Acta Phys. Pol. A.

[82] Gawron W., Bielecki Z., Wojtas J., Stanaszek D., Lach J., Fimiarz M. (2012). Infrared detection module for optoelectronic sensors. Proc. SPIE.

[83] Bielecki Z., Janucki J., Kawalec A., Mikołajczyk J., Palka N., Pasternak M., Pustelny T., Stacewicz T., Wojtas J. (2012). Sensors and systems for the detection of explosive devices. Metrol. Meas. Syst..

[84] Wojtas J., Stacewicz T., Bielecki Z., Rutecka B., Medrzycki R., Mikolajczyk J. (2013). Towards optoelectronic detection of explosives. Opt. Electron. Rev..

[85] Hatab N.A., Eres G., Hatzingerc P.B., Gua B. (2010). Detection and analysis of cyclotrimethylenetrinitramine (RDX) in environmental samples by surface-enhanced Raman spectroscopy. J. Raman Spectrosc..

[86] de la Ossa M.Á.F., Torrea M., García-Ruiza C. (2012). Determination of nitrocellulose by capillary electrophoresis with laser-induced fluorescence detection. Anal. Chim. Acta.

[87] Lucena P., Dona A., Tobaria L.M., Laserna J.J. (2011). New challenges and insights in the detection and spectral identification of organic explosives by laser induced breakdown spectroscopy. Spectrochim. Acta B.

[88] Palka N. (2010). Spectroscopy of explosive materials in the THz range. Acta Phys. Pol. A.

[89] Schubert H., Kuznetsov A. (2005). Detection and Disposal of Improvised Explosives.

[90] Onat B.M., Carver G., Itzler M. (2009). A solid-state hyperspectral imager for real time standoff explosives detection using shortwave infrared imaging. Proc. SPIE.

